# Supplementary material for: Nanomechanical binding mechanism of ligands drives agonistic activity
Source: Nat Commun. 2025 Jul 19;16:6674. doi: 10.1038/s41467-025-61929-1 (PMC12276331; doi:10.1038/s41467-025-61929-1)
Supplement: Supplementary file 1 — Supplementary Information [file 41467_2025_61929_MOESM1_ESM.pdf]

Supplementary Material

of the manuscript

Nanomechanical binding mechanism of ligands drives agonistic activity

Supplementary Tables

Table S1 | Comparison of association rate constants k_{on} and association rates k_2 for double bond formation of hIgG subclasses and hCD40L obtained with SMFS and SPR. The errors of k_{on} and k_2 are standard errors derived from least squares fitting.

	SMFS		SPR	
	**k_{on} [$M^{-1}s^{-1}$]**	**k_2 [s^{-1}]**	**k_{on} [$M^{-1}s^{-1}$]**	**k_2 [$RU^{-1}s^{-1}$]**
**hIgG1**	$2.6 \times 10^{4\ \pm 0.09}$	$5.5 \times 10^{0\ \pm 0.17}$	2.5×10^{4}	5.4×10^{-4}
**hIgG4**	$1.9 \times 10^{4\ \pm 0.08}$	$4.8 \times 10^{0\ \pm 0.14}$	3.6×10^{4}	2.4×10^{-4}
**hIgG2A**	$1.7 \times 10^{4\ \pm 0.04}$	$2.2 \times 10^{0\ \pm 0.13}$	4.1×10^{4}	1.3×10^{-4}
**hIgG2B**	$2.8 \times 10^{4\ \pm 0.08}$	$3.9 \times 10^{0\ \pm 0.15}$	4.4×10^{4}	1.5×10^{-4}
**CD40L**	$1.6 \times 10^{5\ \pm 0.07}$	$1.7 \times 10^{1\ \pm 0.09}$	5.8×10^{4}	1.3×10^{-3}

Table S2 | Summary of dissociation rate constants k_{off} and bond lifetimes $\tau = 1/k_{off}$ of single hIgG:hCD40 and hCD40L:hCD40 bonds derived with the Bell-Evans model and dissociation rates $k_{off,2}$ and bond lifetimes $\tau_2 = 1/k_{off,2}$ for bivalent hIgG:hCD40 and hCD40L:hCD40 bonds calculated with William's Markov model. Errors of k_{off} are standard errors derived from least squares fitting. Other errors were calculated using error propagation.

	**k_{off} [s^{-1}]**	**τ [s]**	**$k_{off,2}$ [s^{-1}]**	**τ_2 [s]**
**hIgG1**	$4.1 \times 10^{-2\ \pm 0.26}$	$2.44 \times 10^{1\ \pm 0.26}$	$2.7 \times 10^{-2\ \pm 0.2}$	$3.66 \times 10^{1\ \pm 0.26}$
**hIgG4**	$4.1 \times 10^{-2\ \pm 0.15}$	$2.44 \times 10^{1\ \pm 0.15}$	$2.7 \times 10^{-2\ \pm 0.15}$	$3.66 \times 10^{1\ \pm 0.15}$
**hIgG2A**	$2.9 \times 10^{-2\ \pm 0.25}$	$3.45 \times 10^{1\ \pm 0.25}$	$1.9 \times 10^{-2\ \pm 0.25}$	$5.17 \times 10^{1\ \pm 0.25}$
**hIgG2B**	$3.0 \times 10^{-2\ \pm 0.16}$	$3.33 \times 10^{1\ \pm 0.16}$	$2.0 \times 10^{-2\ \pm 0.16}$	$5.00 \times 10^{1\ \pm 0.16}$
**CD40L**	$3.5 \times 10^{-1\ \pm 0.10}$	$2.86 \times 10^{0\ \pm 0.10}$	$2.3 \times 10^{-1\ \pm 0.10}$	$4.29 \times 10^{0\ \pm 0.10}$

Supplementary Figures

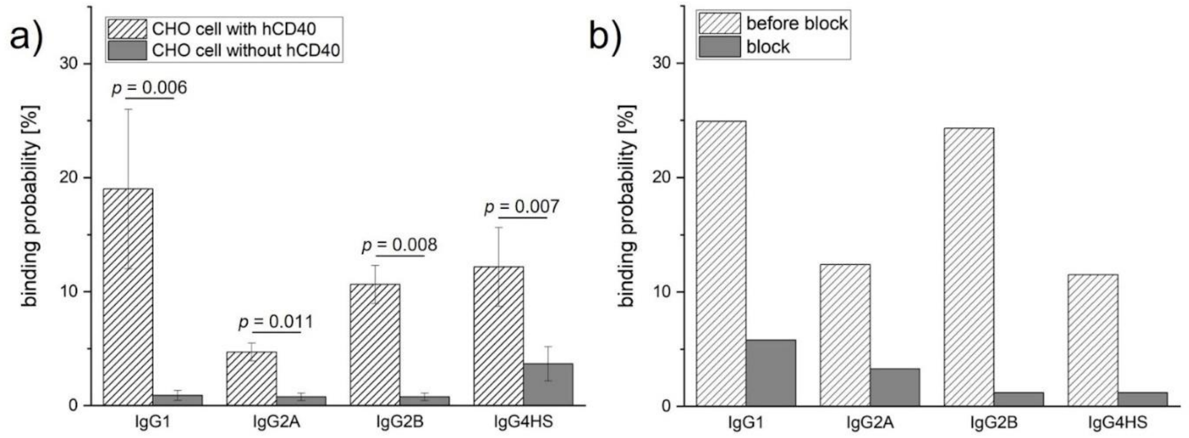

Fig. S1 | SMFS control experiments. Binding probabilities were compared between measurements **(a)** on three different CHO cells with and without hCD40 expression and **(b)** before and after blocking the hCD40 binding paratope of hIgG on the AFM tip with soluble extracellular hCD40. A significant drop ($p < 0.05$) in the binding probability was visible for measurements on cells without hCD40 and after the AFM tip block, showing that signals measured on CHO cells expressing hCD40 do originate from specific hIgG:hCD40 interactions.

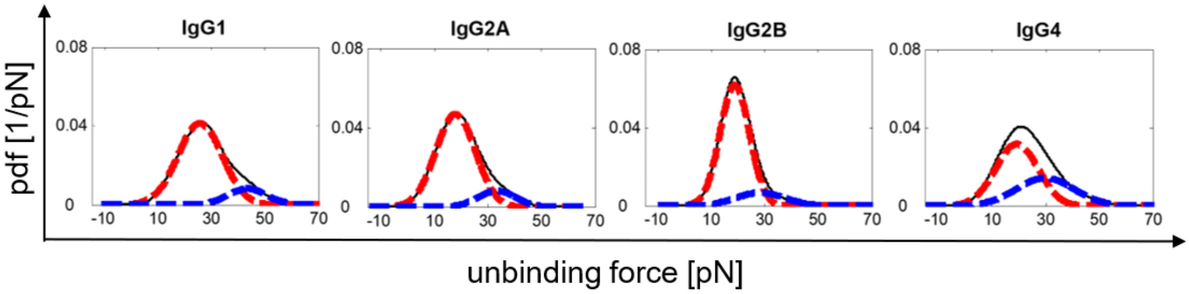

Fig. S2 | Probability density functions (pdfs, black curves) for four different IgG subclasses. The peaks were fitted with Gaussians, summing up unbinding forces of monovalent (red) and bivalent (blue) hIgG:hCD40 bonds.

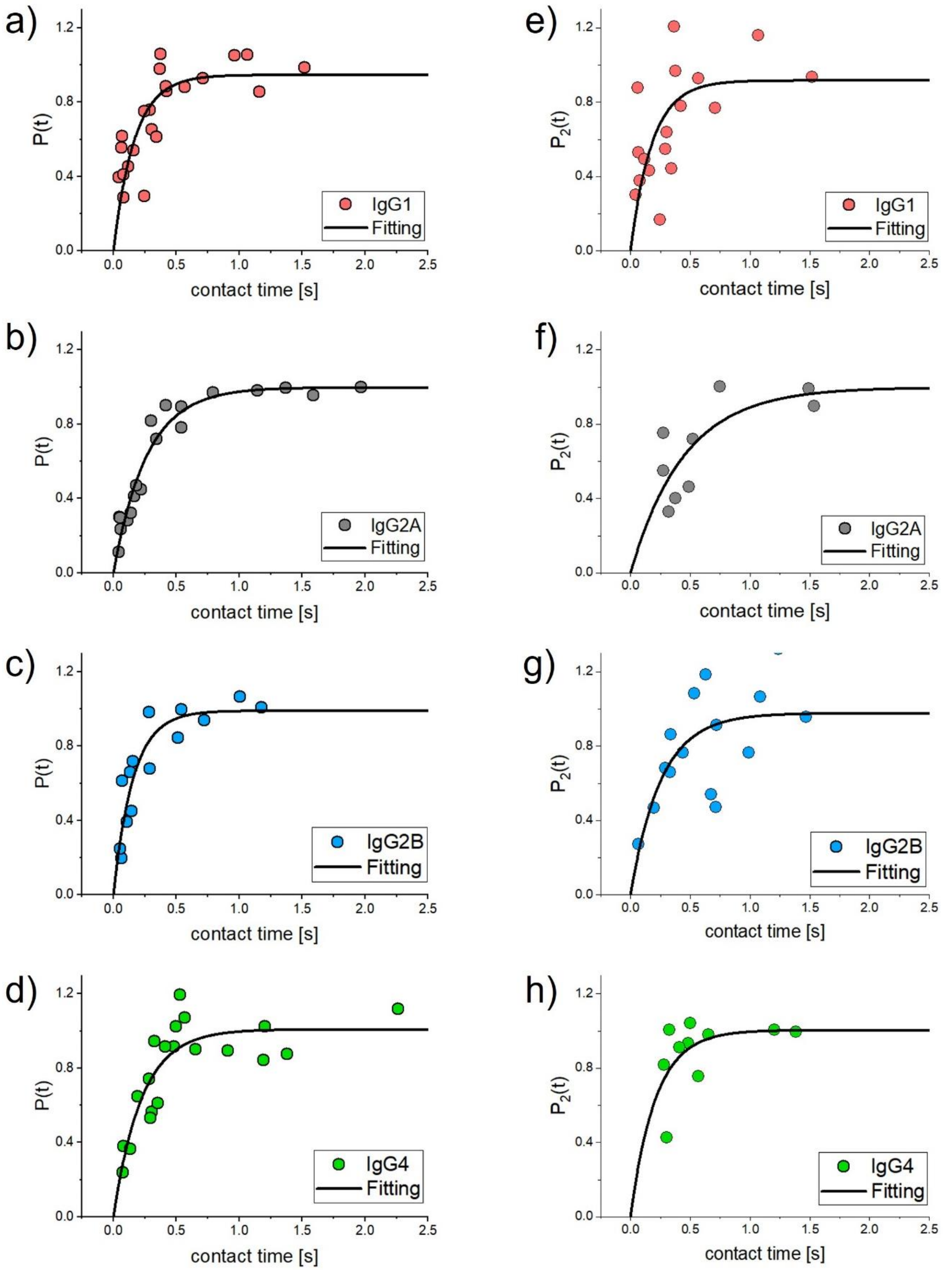

Fig. S3 | Increase of binding probability over contact time measured with SMFS. (a-d) Binding probabilities $P(t)$ of single and **(e-h)** binding probabilities $P_2(t)$ of double hIgG:hCD40 interactions were fitted with $P = A(1\text{-}exp(-(t_c\text{-}t_0)/\tau))$, with A the maximum binding probability, t_0 the lag time and t_c the contact time to further derive kinetic association rate constants for single bonds and kinetic association rates for the formation of a second bonds between hIgG and hCD40.

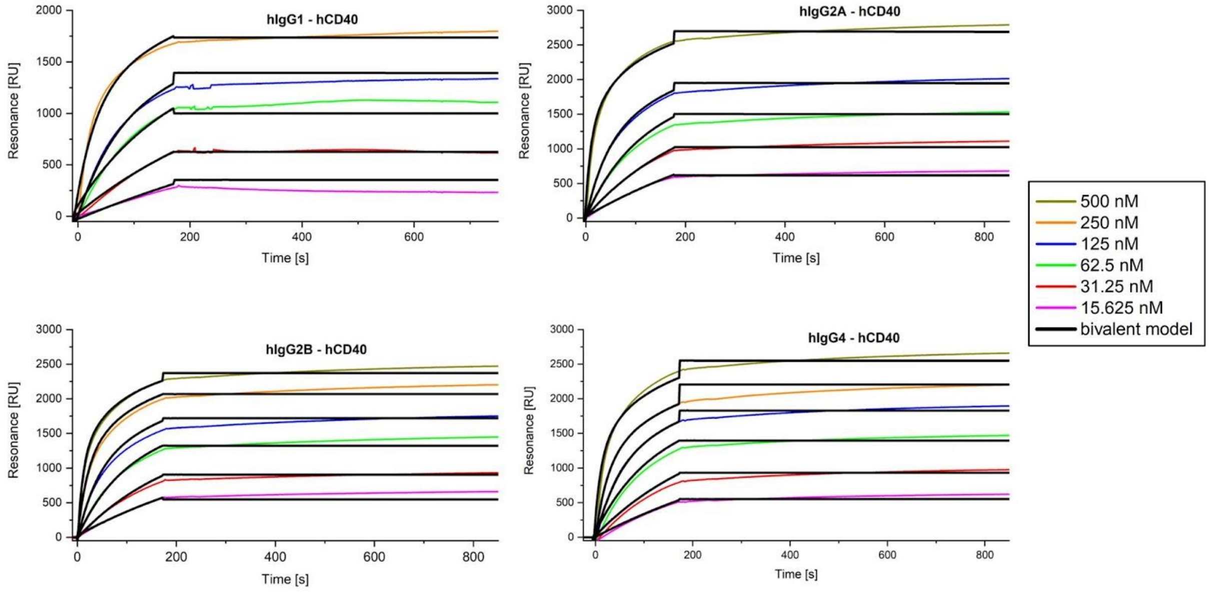

Fig. S4 | Interactions of hIgG subclasses to hCD40 measured with SPR. Association and dissociation kinetics of hIgG subclasses to soluble hCD40 for different hIgG concentrations measured by SPR. Data was fitted with the bivalent binding model (black fit).

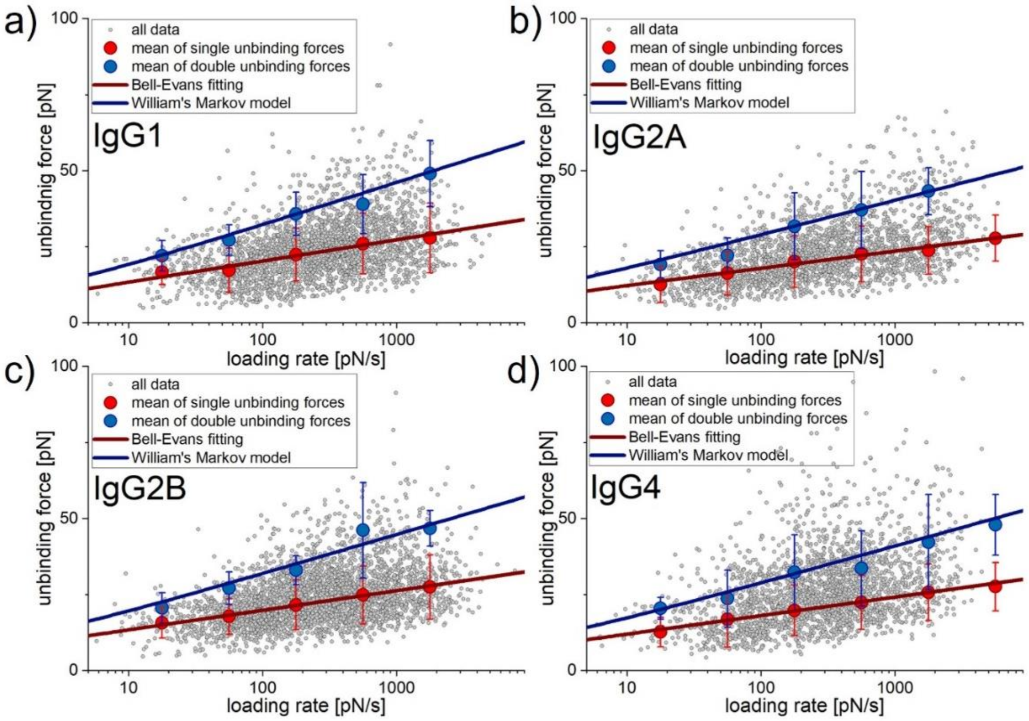

Fig. S5 | Unbinding force versus loading rate plot of different hIgG subclasses. (a-d) Data points (grey) were divided into loading rate segments. Pdfs of unbinding forces of each segment were created and main peaks were fitted with Gaussian curves. The Bell-Evans model (red fit) was applied to the mean and standard deviation of each Gaussian (red dots) to calculate k_{off} and x_B. Experimental data was in good agreement with the William's Markov binding model (blue fit), predicting unbinding forces for two identical parallel bonds.

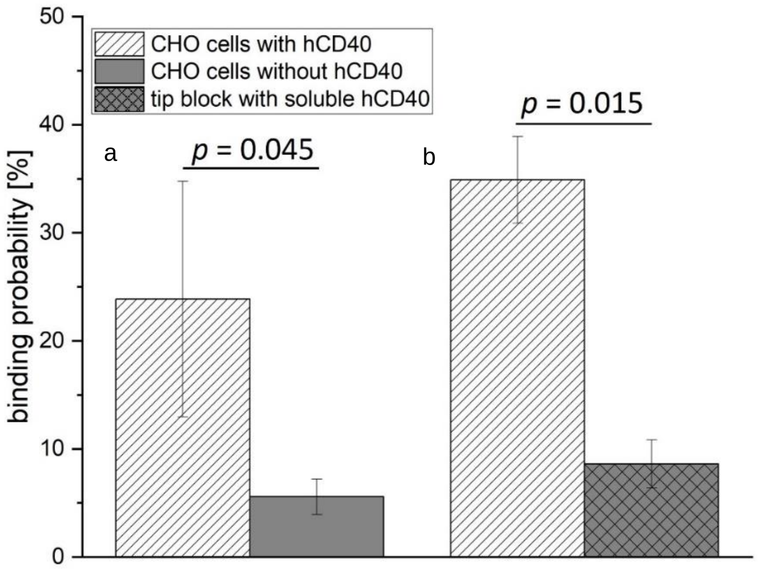

Fig. S6 | SMFS control experiments. Binding probabilities were compared between measurements **(a)** on three different CHO cells with and without hCD40 expression and **(b)** on two different CHO cells before and after blocking the hCD40 binding paratope of hCD40L on the AFM tip with soluble extracellular hCD40. A significant drop ($p < 0.05$) in the binding probability was visible for measurements on cells without hCD40 and after the AFM tip block, showing that signals measured on CHO cells expressing hCD40 do originate from specific hCD40L:hCD40 interactions.

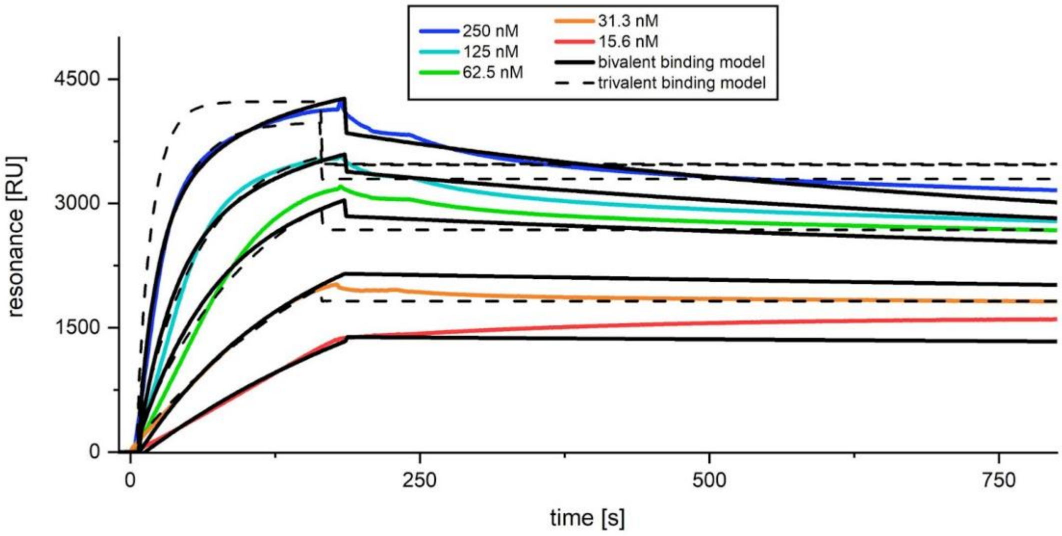

Fig. S7 | Interactions of hCD40L to hCD40 measured with SPR. Association and dissociation kinetics of hCD40L to soluble hCD40 for different CD40L concentrations measured by SPR. Data was fitted with the bivalent binding model (solid black lines) and the trivalent binding model (dashed black lines).
