# Supplementary material for: Nanomechanical binding mechanism of ligands drives agonistic activity
Source: Nat Commun. 2025 Jul 19;16:6674. doi: 10.1038/s41467-025-61929-1 (PMC12276331; doi:10.1038/s41467-025-61929-1)
Supplement: Supplementary file 2 — Description of Additional Supplementary Files [file 41467_2025_61929_MOESM2_ESM.pdf]

**Description of Additional Supplementary Files**

**All movies show raw data from HS-AFM recordings capturing the dynamics of single molecules on a mica surface under physiological conditions.**

**File Name:** Supplementary Movie 1
**Description:** Dynamics of the self-assembly of two hCD40 monomers into a dimer.

**File Name:** Supplementary Movie 2
**Description:** Dynamics of the disassembly of a hCD40 octamer into a dimer and a hexamer.

**File Name:** Supplementary Movies 3 – 4
**Description:** hIgG2B forming monovalent or bivalent bonds with hCD40 assemblies of different sizes. hIgG2B is marked by a y-shaped outline. Purple and magenta circles indicate monovalent and bivalent hIgG2B:hCD40 bonds, respectively. White circles indicate an unbound state.

**File Name:** Supplementary Movies 5 – 6
**Description:** hIgG1 forming monovalent or bivalent bonds with hCD40 assemblies of different sizes. hIgG1 is marked by a y-shaped outline. Purple and magenta circles indicate monovalent and bivalent hIgG1:hCD40 bonds, respectively. White circles indicate an unbound state.

**File Name:** Supplementary Movie 7
**Description:** HS-AFM recordings of hIgG2B showing the individual flexibility of its Fab arms within a certain radius. hIgG2B is marked by a y-shaped outline. Purple circles indicate monovalent hIgG1:hCD40 bonds, white circles indicate an unbound state.

**File Name:** Supplementary Movie 8
**Description:** Dynamics of soluble trimeric hCD40L.

**File Name:** Supplementary Movies 9 – 10
**Description:** Association and dissociation of hCD40 assemblies with hCD40L at three different binding sites, resulting in the formation of monovalent or bivalent bonds. Blue circles indicate hCD40L:hCD40 bonds, white circles indicate an unbound state.

**File Name:** Supplementary Movies 11 – 12
**Description:** Association and dissociation of hCD40L with hCD40 assemblies of various size.

**File Name:** Supplementary Movie 13
**Description:** Large hCD40L:hCD40 clusters of extracellular hCD40 and hCD40L. hCD40 and hCD40L were pre-mixed and incubated for 45 minutes before pipetting onto the mica surface.
