# Supplementary material for: Nanomechanical binding mechanism of ligands drives agonistic activity
Source: Nat Commun. 2025 Jul 19;16:6674. doi: 10.1038/s41467-025-61929-1 (PMC12276331; doi:10.1038/s41467-025-61929-1)
Supplement: Supplementary file 16 — Reporting Summary [file 41467_2025_61929_MOESM16_ESM.pdf]

nature portfolio

Corresponding author(s): Peter Hinterdorfer

Last updated by author(s): May 15, 2025

Reporting Summary

Nature Portfolio wishes to improve the reproducibility of the work that we publish. This form provides structure for consistency and transparency in reporting. For further information on Nature Portfolio policies, see our Editorial Policies and the Editorial Policy Checklist.

Statistics

For all statistical analyses, confirm that the following items are present in the figure legend, table legend, main text, or Methods section.

n/a	Confirmed	
☐	☒	The exact sample size (*n*) for each experimental group/condition, given as a discrete number and unit of measurement
☐	☒	A statement on whether measurements were taken from distinct samples or whether the same sample was measured repeatedly
☐	☒	The statistical test(s) used AND whether they are one- or two-sided<br>*Only common tests should be described solely by name; describe more complex techniques in the Methods section.*
☒	☐	A description of all covariates tested
☒	☐	A description of any assumptions or corrections, such as tests of normality and adjustment for multiple comparisons
☐	☒	A full description of the statistical parameters including central tendency (e.g. means) or other basic estimates (e.g. regression coefficient) AND variation (e.g. standard deviation) or associated estimates of uncertainty (e.g. confidence intervals)
☐	☒	For null hypothesis testing, the test statistic (e.g. *F*, *t*, *r*) with confidence intervals, effect sizes, degrees of freedom and *P* value noted<br>*Give P values as exact values whenever suitable.*
☒	☐	For Bayesian analysis, information on the choice of priors and Markov chain Monte Carlo settings
☒	☐	For hierarchical and complex designs, identification of the appropriate level for tests and full reporting of outcomes
☒	☐	Estimates of effect sizes (e.g. Cohen's *d*, Pearson's *r*), indicating how they were calculated

Our web collection on statistics for biologists contains articles on many of the points above.

Software and code

Policy information about availability of computer code

Data collection	Picoview 1.12 of Agilent 5500 AFM; BiacoreX control software;
Data analysis	Matlab; Origin; Biaevaluation 3.2 RC; Gwyddion 2.56;

For manuscripts utilizing custom algorithms or software that are central to the research but not yet described in published literature, software must be made available to editors and reviewers. We strongly encourage code deposition in a community repository (e.g. GitHub). See the Nature Portfolio guidelines for submitting code & software for further information.

Data

Policy information about availability of data

All manuscripts must include a data availability statement. This statement should provide the following information, where applicable:

- Accession codes, unique identifiers, or web links for publicly available datasets
- A description of any restrictions on data availability
- For clinical datasets or third party data, please ensure that the statement adheres to our policy

Source data are provided with this paper.

Research involving human participants, their data, or biological material

Policy information about studies with human participants or human data. See also policy information about sex, gender (identity/presentation), and sexual orientation and race, ethnicity and racism.

Reporting on sex and gender	*Use the terms sex (biological attribute) and gender (shaped by social and cultural circumstances) carefully in order to avoid confusing both terms. Indicate if findings apply to only one sex or gender; describe whether sex and gender were considered in study design; whether sex and/or gender was determined based on self-reporting or assigned and methods used. Provide in the source data disaggregated sex and gender data, where this information has been collected, and if consent has been obtained for sharing of individual-level data; provide overall numbers in this Reporting Summary. Please state if this information has not been collected. Report sex- and gender-based analyses where performed, justify reasons for lack of sex- and gender-based analysis.*
Reporting on race, ethnicity, or other socially relevant groupings	*Please specify the socially constructed or socially relevant categorization variable(s) used in your manuscript and explain why they were used. Please note that such variables should not be used as proxies for other socially constructed/relevant variables (for example, race or ethnicity should not be used as a proxy for socioeconomic status). Provide clear definitions of the relevant terms used, how they were provided (by the participants/respondents, the researchers, or third parties), and the method(s) used to classify people into the different categories (e.g. self-report, census or administrative data, social media data, etc.) Please provide details about how you controlled for confounding variables in your analyses.*
Population characteristics	*Describe the covariate-relevant population characteristics of the human research participants (e.g. age, genotypic information, past and current diagnosis and treatment categories). If you filled out the behavioural & social sciences study design questions and have nothing to add here, write "See above."*
Recruitment	*Describe how participants were recruited. Outline any potential self-selection bias or other biases that may be present and how these are likely to impact results.*
Ethics oversight	*Identify the organization(s) that approved the study protocol.*

Note that full information on the approval of the study protocol must also be provided in the manuscript.

Field-specific reporting

Please select the one below that is the best fit for your research. If you are not sure, read the appropriate sections before making your selection.

☒ Life sciences ☐ Behavioural & social sciences ☐ Ecological, evolutionary & environmental sciences

For a reference copy of the document with all sections, see nature.com/documents/nr-reporting-summary-flat.pdf

Life sciences study design

All studies must disclose on these points even when the disclosure is negative.

Sample size	Force measurements for each type of IgG and CD40L were performed on at least two different cells. For every cell, force distance curves at six different pulling velocities were measured. For each velocity at least 500 curves were measured.
Data exclusions	For Bell Evans' fitting, individual binding forces were plotted versus their loading rate. From there, the forces were divided into several loading rate segement and probability density functions of unbinding forces of each segment were fitted with the sum of two Gaussians. The mean and standard deviation of the first Gaussian was plotted as a function of the loading rate and fitted using the Bell-Evans model.
Replication	For force measurements, each type of IgG and CD40L was tested on at least two cells. The results on these cells are replicable. In addition, control experiments for each type of IgG and CD40L were performed to verify the specificity of the recorded force signals.
Randomization	Only cells with very low CD40-GFP expression were excluded from force measurements. Otherwise the selection of cells was random.
Blinding	The investigations were blinded, as we collected all measured data and obtained results from data analysis.

Reporting for specific materials, systems and methods

We require information from authors about some types of materials, experimental systems and methods used in many studies. Here, indicate whether each material, system or method listed is relevant to your study. If you are not sure if a list item applies to your research, read the appropriate section before selecting a response.

Materials & experimental systems

n/a	Involved in the study	
[ ]	[x]	Antibodies
[ ]	[x]	Eukaryotic cell lines
[x]	[ ]	Palaeontology and archaeology
[x]	[ ]	Animals and other organisms
[x]	[ ]	Clinical data
[x]	[ ]	Dual use research of concern
[x]	[ ]	Plants

Methods

n/a	Involved in the study	
[x]	[ ]	ChIP-seq
[x]	[ ]	Flow cytometry
[x]	[ ]	MRI-based neuroimaging

Antibodies

Antibodies used

hIgG1, hIgG2A, hIgG2B and hIgG4 of anti-CD40 antibody ChiLob 7/4 (Pfizer Inc., USA)

Validation

Binding of each type of human IgG to human CD40 was tested via single molecule force spectroscopy and surface plasmon resonance and visualized by high-speed AFM, as described in the manuscript. The antibody was investigated in other publications:

Geldart et al. Cancer immunotherapy with a chimeric anti-CD40 monoclonal antibody: Evidence of preclinical efficacy. Journal of Clinical Oncology 22, 2577–2577 (2004).

Johnson, P. et al. Clinical and Biological Effects of an Agonist Anti-CD40 Antibody: A Cancer Research UK Phase I Study. Clinical Cancer Research 21, 1321–1328 (2015).

Eukaryotic cell lines

Policy information about cell lines and Sex and Gender in Research

Cell line source(s)

CHO (Chinese hamster ovary) cells were transfected with Lipofectamine 2000 (Thermo Fisher Scientific Inc., #11668027) using a CD40 green fluorescent protein (GFP) fusion construct (Origene, #RG201977) to yield CHO-CD40-GFP cells.

Authentication

The cell line was not further authenticated for force measurements

Mycoplasma contamination

The cell line had no mycoplasma contamination. It was not further examined for force measurements.

Commonly misidentified lines
(See ICLAC register)

No
